# Genes Important for *Schizosaccharomyces pombe* Meiosis Identified Through a Functional Genomics Screen

**DOI:** 10.1534/genetics.117.300527

**Published:** 2017-12-19

**Authors:** Julie Blyth, Vasso Makrantoni, Rachael E. Barton, Christos Spanos, Juri Rappsilber, Adele L. Marston

**Affiliations:** * Wellcome Centre for Cell Biology, Institute of Cell Biology, School of Biological Sciences, University of Edinburgh, EH9 3BF, UK; † Institute of Biotechnology, Technische Universität Berlin, 13355 Berlin, Germany

**Keywords:** meiosis, *Schizosaccharomyces pombe*, sporulation, chromosome segregation

## Abstract

Meiosis is a specialized cell division that generates gametes, such as eggs and sperm. Errors in meiosis result in miscarriages and are the leading cause of birth defects; however, the molecular origins of these defects remain unknown. Studies in model organisms are beginning to identify the genes and pathways important for meiosis, but the parts list is still poorly defined. Here we present a comprehensive catalog of genes important for meiosis in the fission yeast, *Schizosaccharomyces pombe*. Our genome-wide functional screen surveyed all nonessential genes for roles in chromosome segregation and spore formation. Novel genes important at distinct stages of the meiotic chromosome segregation and differentiation program were identified. Preliminary characterization implicated three of these genes in centrosome/spindle pole body, centromere, and cohesion function. Our findings represent a near-complete parts list of genes important for meiosis in fission yeast, providing a valuable resource to advance our molecular understanding of meiosis.

SEXUALLY reproducing organisms rely on meiosis to generate gametes with half the number of chromosomes of the parental cell. Following DNA replication, two consecutive nuclear divisions occur. During meiosis I, the maternal and paternal chromosomes, called homologs, are segregated to opposite poles. However, sister chromatids are segregated away from each other only during meiosis II, in a manner resembling chromosome segregation during mitosis. In addition to an alteration in cell cycle controls, which allows two consecutive chromosome segregation events to occur after DNA replication, meiosis requires three key modifications to chromosome behavior (reviewed in Marston and Amon (2004)). First, homologs must be linked to ensure their equal partitioning to opposite poles. Typically, meiotic recombination provides these links by generating cross-overs which mature into the chiasmata that physically connect homologs (reviewed in Hunter 2015). Without these links, homologs cannot align and properly segregate during meiosis; therefore, meiotic recombination is essential for proper chromosome segregation during meiosis I. Second, the cosegregation of sister chromatids during meiosis I requires that the sister kinetochores are held together. In budding yeast, maize, and flies, sister kinetochore fusion is thought to underlie sister chromatid comigration (Goldstein 1981; Li and Dawe 2009; Sarangapani *et al.* 2014). However, sister kinetochores are frequently observed to be individualized in human oocytes, potentially contributing to the high level of aneuploidy observed (Patel *et al.* 2015; Zielinska *et al.* 2015). Third, the cohesion that is established between sister chromatids during DNA replication is lost in two steps during meiosis. During meiosis I, the meiosis-specific cohesin complex component, Rec8, is cleaved on chromosome arms, triggering the segregation of homologs to opposite poles (Watanabe and Nurse 1999; Buonomo *et al.* 2000). However, cohesin is protected in the region surrounding the centromere, called the pericentromere, during meiosis I. Maintenance of pericentromeric cohesin during meiosis I is critical for the accurate segregation of sister chromatids during meiosis II and relies on the conserved complex of shugoshin and protein phosphatase 2A (reviewed in Marston 2015).

Over the last two decades, a variety of approaches using a multitude of model organisms have led to the identification of key factors important for meiosis. For example, a recent mutagenesis screen in *Arabidopsis thaliana* identified *MTOPVIB* (Vrielynck *et al.* 2016), the essential B subunit of the meiotic double-strand break Topoisomerase VI-like endonuclease, Spo11, which was discovered 20 years ago in *Saccharomyces cerevisiae* through a biochemical approach (Keeney *et al.* 1997). Elegant screens designed to target a particular process identified shugoshin (Kitajima *et al.* 2004), the meiosis I-regulator Moa1 in *Schizosaccharomyces pombe* (Yokobayashi and Watanabe 2005), and components of the monopolin complex required for sister kinetochore coorientation during meiosis I (Rabitsch *et al.* 2003). Systematic knockout screens of genes upregulated during meiosis identified the founding member of monopolin in budding yeast (Toth *et al.* 2000; Rabitsch *et al.* 2001), shugoshin in fission yeast (Rabitsch *et al.* 2004), and Meikin, the functional homolog of fission yeast Moa1, in mouse (Kim *et al.* 2015). Our previous genome-wide functional screen using the *S. cerevisiae* deletion library identified shugoshin and kinetochore proteins important for centromere cohesion (Marston *et al.* 2004). We recently elucidated the molecular basis by which these kinetochore proteins target cohesin to centromeres (Hinshaw *et al.* 2015, 2017).

Budding yeast *S. cerevisiae* and fission yeast *S. pombe* are particularly useful discovery tools as their ability to propagate as haploids and the availability of deletion libraries representing the entire genome mean comprehensive genome-wide screens are eminently feasible. Both yeasts exist as haploids with two mating types that can undergo conjugation, followed by nuclear fusion (karyogamy); and the resultant diploids enter meiosis, eventually differentiating into four haploid ascospores, collectively called a tetrad. Moreover, *S. cerevisiae* and *S. pombe* are separated by ∼350 MY of evolution and therefore provide complementary insight. *S. pombe* screens monitoring the ability of each mutant to form spore coats have revealed that ∼10 or ∼1% of genes are required for sporulation in *S. cerevisiae* and *S. pombe*, respectively (Enyenihi and Saunders 2003; Ucisik-Akkaya *et al.* 2014). However, many mutants with impaired chromosome segregation are not defective in spore formation, and therefore these screens will fail to identify many factors important for meiosis.

Here, we have adapted and improved the procedure we previously used to screen the *S. cerevisiae* deletion library (Marston *et al.* 2004) for *S. pombe*, allowing us to provide a comprehensive list of virtually all nonessential genes important for meiosis and sporulation in this organism. We identified 354 genes that are important for efficient spore formation, suggesting roles in conjugation, meiotic entry or division, and asci formation. Only 12% of these have been previously implicated in meiosis, sporulation, or conjugation and 13% are of unknown function. We also found 269 genes important for accurate chromosome segregation, of which 15% were uncharacterized. As proof of principle, we performed preliminary characterization of the roles of three genes in meiosis and sporulation. First, we show that *acb1*, encoding a predicted acyl-CoA binding protein, contributes to ensuring robust sister chromatid cohesion and proper levels of meiotic recombination. Second, we establish an important role for the F box protein, Pof3, in chromosome segregation during both meiosis I and meiosis II. Third, we identify *SPCC1739.04c*, which we name *dms1* (for *defective meiosis and sporulation 1*), as encoding a novel spindle pole body associated protein, critical for spore formation. This screen provides a valuable resource to direct future studies on meiosis and sporulation.

## Materials and Methods

### Yeast strains and culture

Haploid *S. pombe* deletion set v2.0 containing 3004 strains, each with a deletion in a single nonessential gene, was supplied by Bioneer Corporation (Kim *et al.* 2010). A further 281 deletion strains were generated by the Gould laboratory (Chen *et al.* 2014). We were unable to revive 131 strains from the glycerol stock. Additional *S. pombe* strains used are listed in Supplemental Material, Tables S4 and Table S8 in File S1. Standard media and growth conditions were used unless otherwise stated. Synchronous meiosis of adenine-prototrophic diploids was performed as previously described (Loidl and Lorenz 2009). Genes were deleted or tagged using a PCR-based method as previously described (Bahler *et al.* 1998).

### Genetic screen

A query strain AMfy85 was generated with three key elements. First, a *NatMX6* marker was integrated adjacent to h^90^ in the homothallic strain so that following selection of haploids and mating type switching, diploids that can undergo meiosis and sporulation are produced. Second, *lacI-GFP-leu2+* and *lacO-ura4+* arrays were integrated on the arm of chromosome II. Third, the query strain carried the *rpl42P56Q* allele, which confers recessive cycloheximide-resistance (cyh^R^), allowing counterselection against heterozygous diploids. Each strain from the haploid Bioneer collection V2 was crossed to the query strain and a series of selection steps was used to select the h^90^ allele and GFP-marked chromosomes. Briefly, the query strain was mixed with each library strain on sporulation agar (SPA) plates and incubated at 25° for 5 days. The patch was then transferred to YES plates containing G418 and CloNat for 2 days at 30°, then pombe minimal glutamate (PMG) plates with cycloheximide lacking leucine and uracil. After 2 days, patches were transferred to YES at 30° overnight and finally onto SPA plates for 2 days at 25° prior to analysis. All steps were performed in the dark to ensure integrity of the GFP signal and adenine was added in excess to reduce background fluorescence.

### Microscopy

Strains for the homozygous GFP-labeled chromosome assays (except during the full Bioneer screen as above) were grown on a YES plate for 2–3 days at 25° then transferred to an SPA plate at 25° for 2 days. For the heterozygous GFP-labeled chromosome assay, each strain was grown in liquid YES to mid log phase, washed in PM-N, combined with the opposite mating type, and incubated on an SPA plate for 18 hr at 25°. Cells (including the full Bioneer screen) were applied to a glass slide with water. To visualize the DNA, cells were fixed in ice-cold methanol for 1 min, then applied to a slide. One microgram per milliliter of DAPI was added and cells were imaged on a Zeiss Axioplan 2 microscope with a 100× Plan ApoChromat NA 1.4 oil lens.

Strains for live cell imaging were grown to log phase in liquid PMG plus supplements, transferred to an SPA plate and incubated for 18 hr at 18° and resuspended in PM-N before mounting (for strains containing the h^90^ mating type). For imaging diploids, a sample was removed 4 hr after nitrogen starvation. Cells were mounted on glass bottom dishes (either a MatTek Corporation 35-mm glass bottom dish or Ibidi 15-μm four- or eight-well glass bottom slide coated with lectin). Images were acquired at 25° on a temperature-controlled DeltaVision Elite system (Applied Precision, Isaaquah, Washington) using an inverted Olympus IX-71 microscope with a 100× UPlanSApo NA 1.4 oil lens and a Photometrics Cascade II EMCCD camera, operated through SoftWorx software (Applied Precision). Images were processed using ImageJ version 1.59m9 and a maximum projection of 11 Z -sections spaced at 0.4-μm intervals is shown.

### Rec8 intensity measurements

Corrected total cell fluorescence (CTCF) of Rec8-GFP was determined as described by McCloy *et al.* (2014) using the formula CTCF = integrated density − (area of selected cell × fluorescence of background readings) in ImageJ.

### Western blotting, immunoprecipitation, and mass spectrometry

Western blotting was as described by Clift *et al.* (2009). Mouse anti-GFP (Roche) and anti-Tat1 (gift from Keith Gull) antibodies were used at 1:1000. Immunoprecipitation was performed as described by Fox *et al.* (2017) with minor adaptations. Briefly, cells were harvested and washed in sterile water by centrifugation at 4000 rpm for 8 min, resuspended in 0.2× cell volume of sterile water plus 2 mM PMSF and frozen in liquid nitrogen. Cell pellets (3 g) were ground in a Retsch Mixer Mill MM400, thawed in Hyman buffer [50 mM Bis-Tris propane, pH 7; 100 mM KCl; 5 mM EGTA; 10% (v/v Glycerol)] plus inhibitors [5 μg/ml each chymostatin, leupeptin, antipain, pepstatin A, E-64; 4 mM AEBSF (pefablock); 2 mM benzamidine, 2 mM PMSF, 0.4 mM LR-microcystin, 2 mM N-ethylmaleimide (NEM), 0.8 mM sodium orthovanadate, 4 mM β-glycerophosphate, 2 mM sodium pyrophosphate]. Triton X-100 was added to 0.1% and the extract was sonicated at 38% for 1 × 30 sec before spinning at 4000 rpm for 10 min. The supernatant was filtered through a 1.6 μm glass microfiber filter (Whatman 25 mm GD/X) and the cleared extract was added to GFP-TRAP_M (Chromotek) at a ratio of 25 μl beads per ml of extract before incubating at 4° rotating for 1 hr. The beads were washed once with cold Hyman buffer then three times with high-salt Hyman buffer (500 mM KCl). Proteins were eluted by incubating beads with 0.2 M glycine pH 2.5 for 10 min at room temperature with rotation. The supernatant was transferred to a fresh tube containing 1/10th volume 1 M Tris pH 9.0 to neutralize. NuPAGE LDS with 5% β-mercaptoethanol was added, the samples boiled at 100° for 5 min, spun down at 13,000 rpm for 5 min, and loaded onto a precast NuPAGE 8–12% Bis-Tris gel (Thermo Fisher Scientific, Waltham, MA). A Pierce silver staining kit (Thermo Fisher Scientific) was used to visualize bands. For mass spectrometry, excised gel bands were destained and proteins were digested with trypsin, as described by Shevchenko *et al.* (1996). In brief, proteins were reduced in 10 mM dithiothreitol (Sigma Aldrich, St Louis, MO) for 30 min at 37° and alkylated in 55 mM iodoacetamide (Sigma Aldrich) for 20 min at ambient temperature in the dark. Proteins were digested overnight at 37° with 12.5 ng/μl trypsin (Thermo Fisher), diluted with an equal volume of 0.1% TFA and spun onto StageTips as described by Rappsilber *et al.* (2003). Peptides were eluted in 20 μl of 80% acetonitrile in 0.1% TFA and concentrated to 4 μl by vacuum centrifugation (Concentrator 5301, Eppendorf, Hamburg, Germany). The peptide sample was prepared for LC-MS/MS analysis by diluting it to 5 μl by 0.1% TFA. LC-MS-analyses were performed on an Orbitrap Fusion Lumos Tribrid Mass Spectrometer (Thermo Fisher Scientific) coupled online to an Ultimate 3000 RSLCnano System (Thermo Fisher Scientific). Peptides were separated on a 50-cm EASY-Spray column (Thermo Fisher Scientific), which was assembled on an EASY-Spray source (Thermo Fisher Scientific) at 50°. Mobile phase A consisted of 0.1% formic acid in LC-MS grade water and mobile phase B consisted of 80% acetonitrile and 0.1% formic acid. Peptides were loaded onto the column at a flow rate of 0.3 μl/min and eluted at a flow rate of 0.2 μl/min according to the following gradient: 2–40% mobile phase B in 150 min and then to 95% in 11 min. Mobile phase B was retained at 95% for 5 min and returned back to 2% a minute after until the end of the run (190 min). FTMS spectra were recorded at 60,000 resolution (scan range 350–1500 m/z) with an ion target of 7.0e5. MS2 was performed in the orbitrap (30,000 resolution) with ion target of 1.0E4 and HCD fragmentation (Olsen *et al.* 2007) with normalized collision energy of 25. The isolation window in the quadrupole was 1.6 Thomson. Only ions with charge between 2 and 7 were selected for MS2. The MaxQuant software platform (Cox and Mann 2008) version 1.5.2.8 was used to process the raw files and search was conducted against *S. pombe* complete/reference proteome set of PomBase database (released on 01/07/2016), using the Andromeda search engine (Cox *et al.* 2011). For the first search peptide tolerance was set to 20 ppm while for the main search peptide tolerance was set to 4.5 ppm. Isotope mass tolerance was 2 ppm and maximum charge was set to 7. Digestion mode was set to specific with trypsin allowing a maximum of two missed cleavages. Carbamidomethylation of cysteine was set as fixed modification. Oxidation of methionine was set as variable modification. Peptide and protein identifications were filtered to 1% FDR. Mass spectrometry data are available at https://www.ebi.ac.uk/pride/archive/projects/PXD008245.

### Spore viability assay

Spore viability was performed as previously described by Le *et al.* (2013) with minor modifications. Strains were mated on SPA for 48–72 hr. Asci were treated with 1:500 glusulase (Perkin Elmer, Waltham, MA) and incubated at room temperature overnight. Spore count was determined by a hemocytometer and 2000, 5000, or 10,000 spores of wild type, *rec12Δ*, or mutant, respectively, were plated over 10 YES plates. Spore viability was determined as the number of colonies formed over the number of plated spores. Spore viability was normalized to levels observed for the wild type.

### Recombination assay

Genetic recombination was scored as previously described by Davis and Smith (2006). Briefly, wild type and mutant strains were mixed onto SPA plates and incubated for 2 days at 25°. Following overnight digestion in glusulase to inactivate vegetative cells, the surviving spores were plated on YES medium at a density of 1000 spores/ml and incubated at 30° for a further 3 days. Colonies were replica plated onto YE-LA (10 mg/liter instead of 450 mg/liter adenine), PMG-his, and PMG-leu selective plates and allowed to grow for another 1–2 days at 30°. Viable colonies on PMG − leu +his and PMG + leu −his (recombinants) were scored and recombination efficiency was measured as a percentage of recombinants to total colonies that grew on YE–LA plates. Gene conversion was determined by scoring the percentage of total colonies that grew on plates with low adenine. Between 234 and 1443 colonies per cross were scored and six biological repeats were performed.

### GO term analysis

Gene Ontology (GO) enrichment analysis was performed using the Generic GO term finder within the Bioinformatics Group at the Lewis-Sigler Institute, Princeton University (http://go.princeton.edu/cgi-bin/GOTermFinder); GO Slim terms were identified using the Generic GO term mapper (http://go.princeton.edu/cgi-bin/GOTermMapper).

### Data availability

*S. pombe* strains listed in Tables S4 and Table S8 in File S1 are available on request, without restriction. Raw mass spectrometry data are available on PRIDE https://www.ebi.ac.uk/pride/archive/projects/PXD008245.

## Results

### A functional genomic screen identifies factors important for meiosis and sporulation

**Figure 1:**
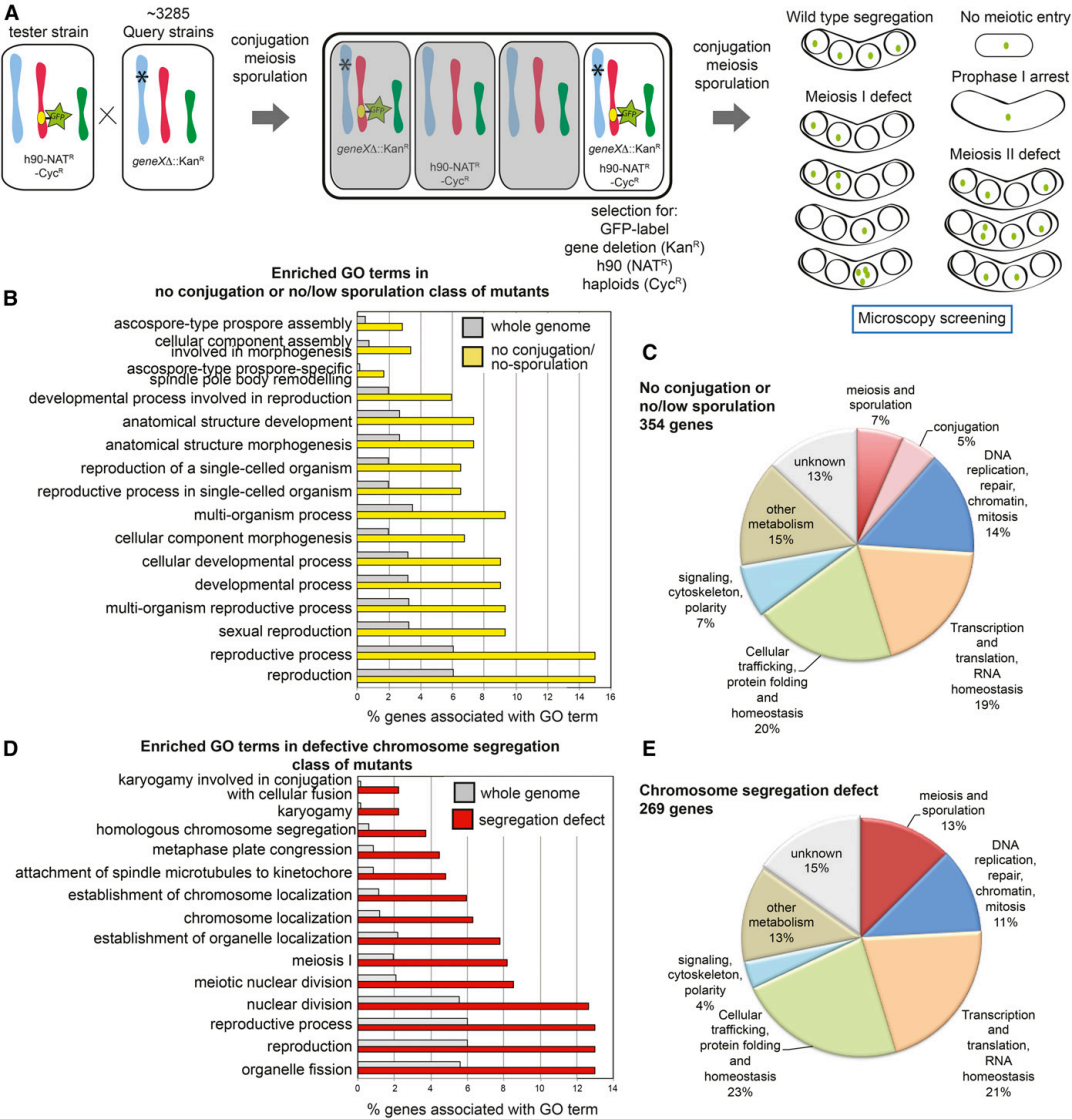
Overview of the functional genomics screen to identify genes important for meiosis and sporulation in *S. pombe*. (A) Screen strategy for systematic analysis of mutants. The tester strain carried: the homothallic *h^90^* locus, allowing mating type switching, linked to the Nourseothricin (NAT)-resistance cassette *NATMX6*; *rpl42P56Q*, which confers recessive cycloheximide-resistance (cyh^R^), allowing counterselection against heterozygous diploids; and a GFP label (*lacO-ura3^+^* and *lacI-GFP-leu2^+^*) to follow segregation of chromosome II. The query strain is one of 3285 library strains in which a single nonessential gene has been replaced by *KANMX6*, conferring resistance to G418. Query and tester strains were mated and allowed to sporulate. Selection for G418-, nourseothricin-, and cycloheximide-resistant leucine and uracil prototrophs allowed the isolation of haploids of mixed mating types carrying the deletion of interest and the GFP-labeled chromosome. These haploids were allowed to mate and sporulate. Meiosis in the absence of a single gene product was assessed visually by scoring the fate of the homozygous GFP label on chromosome II under the microscope. Example patterns of GFP dots in the tetrads are shown. (B) Significantly enriched GO terms associated with the 354 genes whose absence resulted in <20% of cells producing spores in our screen. The percentage of genes fitting this criterion is shown compared to the genome as a whole for each of the GO terms found to be significantly enriched in the no conjugation or low/no sporulation data set. (C) Functional classification of the 354 genes whose absence resulted in <20% sporulation in our screen based on GO terms. The criteria for inclusion in this category was reduced spore formation; therefore the mutant phenotype observed includes mutants that failed to conjugate, enter meiosis, or form asci in the screen. (D) Significantly enriched GO terms associated with the 269 genes whose absence resulted in <90% of tetrads receiving a single GFP dot in each spore. The percentage of genes fitting this criterion is shown compared to the genome as a whole for each of the GO terms found to be significantly enriched in the chromosome segregation defect data set. Note that mutants that produced <20% spores (as shown in C) were not included in this analysis even if they were found to have a chromosome segregation defect. (E) Functional classification based on GO terms of the 269 genes whose absence resulted in a chromosome segregation defect (<90% tetrads with each spore receiving a single GFP dot) in our screen.

To identify genes important for meiosis and sporulation in *S. pombe* we screened a deletion library representing all nonessential genes. Through a mating and selection procedure (Figure 1; *Materials and Methods*) we generated strains that were homozygous for each deletion and where both copies of chromosome II were labeled with GFP. The effect of each gene deletion on meiotic chromosome segregation and sporulation was visually assayed by microscopy. Following normal meiosis and sporulation, only tetrads with a GFP label in each spore (gamete) should be produced. Any deviation from this pattern is suggestive of a defect at some stage (Figure 1A). We screened 3285 strains in this way and performed two analyses. First, we quantified sporulation efficiency by scoring the percentage of cells that formed tetrads by light microscopy. Second, we determined chromosome segregation fidelity by scoring the patterns of GFP dot segregation in those strains that formed tetrads. The analysis for all mutants is given in Figure S1A and Table S1 in File S1. Using the phenotypes displayed by previously identified meiosis and sporulation genes as a guide, we set the “no/low sporulation” cut-off as <20% sporulation and the “chromosome segregation defect” cut-off as <90% tetrads with a single GFP focus in each spore. Mutants that failed to generate spores include those failed in conjugation, meiotic entry, nuclear division, or asci formation and could not always be easily distinguished in the screen. However, in some cases, a high frequency of haploid-like straight cells that did not show the characteristic curved shape of zygotic meiosis suggested that conjugation did not occur; these mutants were denoted as “no conjugation” (see also notes column in Table S1 in File S1 for descriptions of specific mutants). Based on the above criteria we identified 354 mutants with no conjugation, low or no sporulation and 269 genes important for chromosome segregation during meiosis (Figure 1, B–E). GO term analysis (see *Materials and Methods*) revealed significant enrichment of terms associated with reproduction and spore formation for the no/low sporulation class of mutants (Figure 1B) and with nuclear division, chromosome function, and karyogamy for the chromosome segregation defective mutants (Figure 1D), confirming success at identifying the expected functional categories. Guided by the associated GO Slim terms, we sorted genes from each screening method into broad functional categories (Figure 1, C and E and Table S2 and Table S3 in File S1). Only a small percentage (12–13%) had been previously implicated in conjugation, meiosis, or sporulation, and a similar fraction were associated with DNA repair, replication, chromatin, or mitosis. Gene expression and protein homeostasis were also predominantly featured in the list of identified genes. In addition, we identified 13 and 15% of genes in the low/no sporulation and defective chromosome segregation classes, respectively, which were not previously ascribed to a particular cellular process (unknown). Therefore, our functional genomics screen was successful at identifying both known and novel genes important for meiosis and sporulation.

### Verification of screen candidates

To validate genes identified as being important for meiosis in our screen, we selected 100 genes that had been associated with a broad range of functional processes. These genes included some known to be important for mitosis (*e.g.*, *dad2*, *dad3*, *spc19*, members of the DASH kinetochore complex; Buttrick and Millar 2011) as well as many uncharacterized genes (Figure S1, B–D and Table S5 and S6 in File S1). To ensure that the observed phenotypes were due to the annotated gene, we made new knockouts in a strain carrying GFP-labeled chromosome II and scored sporulation efficiency and chromosome segregation (by examining GFP foci pattern or nuclear morphology; Table S4 in File S1). The majority of genes (70%) were confirmed as being important for meiosis and sporulation, as defined by our cut-offs, while we failed to generate 4% of our selected mutants. Multiple reasons could explain why 26% of mutants showed no strong phenotype in our verification screen, for example additional or absent mutations in the parental library strain or inefficient passage through the mating and selection procedure.

**Figure 2:**
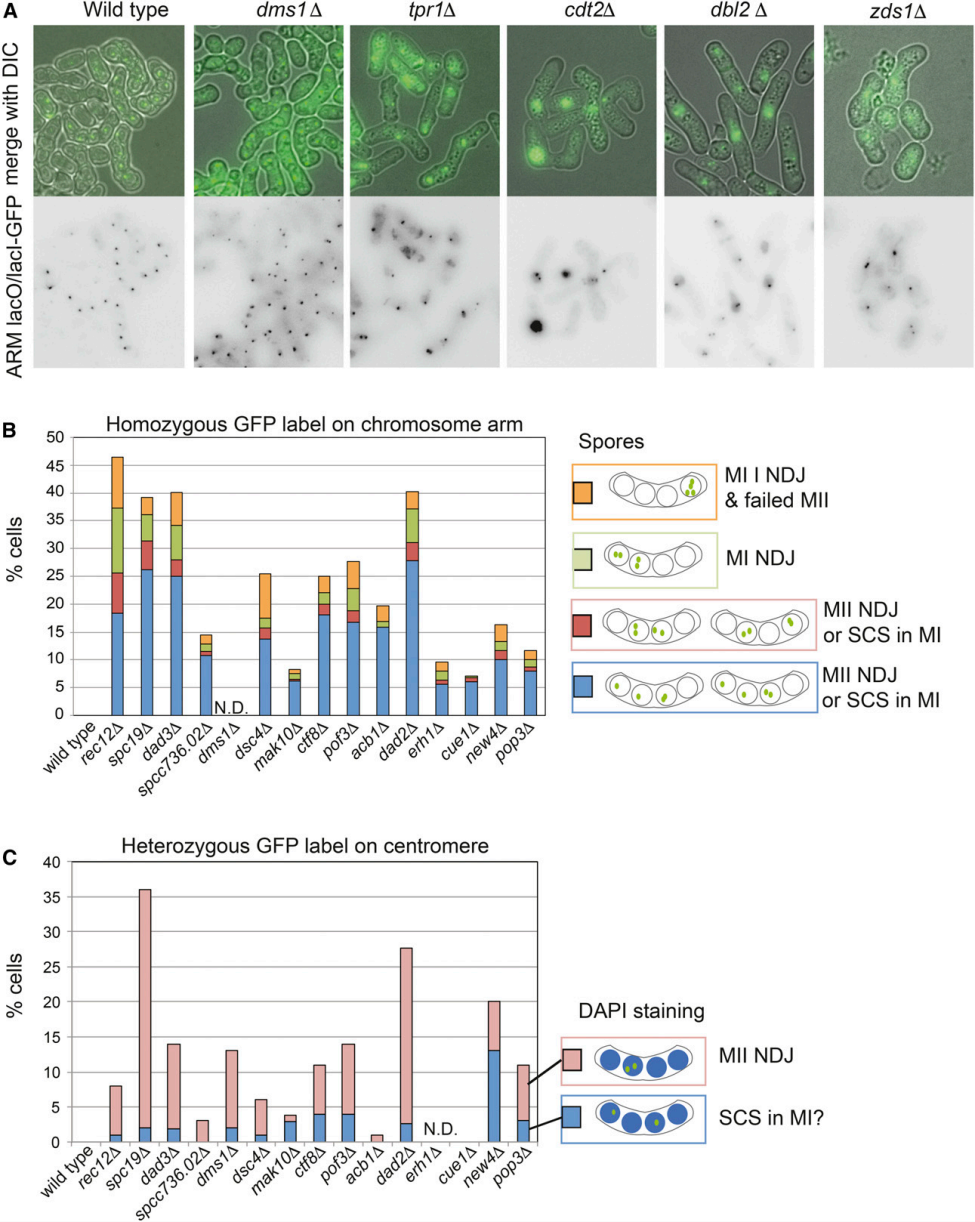
Preliminary characterization of genes important for meiosis. To validate hits in the screen, 18 genes were chosen for further analysis. (A and B) The pattern of GFP foci in tetrads carrying homozygous *lacO/LacI-GFP* on chromosome II was examined after induction of zygotic meiosis. Strains were homothallic (h90), carried the deletion of interest, and were homozygous for *lacO/*LacI-GFP on chromosome II. (A) Representative images of mutants in the no conjugation/no sporulation class, which includes mutants that fail to conjugate (*dbl2Δ*), enter meiosis (*cdt2Δ*, *zds1Δ*), or form asci (*e.g.*, *tpr1Δ*, *dms1Δ*) and a wild type example. Objective used for magnification was ×100. (B) Patterns of segregation of homozygous *lacO/LacI-GFP* on chromosome II observed after zygotic meiosis of homothallic (h^90^) strains with the indicated genotypes. *n=*100; N.D., not determined (*dms1Δ* does not form spores). (C) The pattern of heterozygous *lacO/*LacI-GFP foci integrated close to *cen2* was scored in tetranucleate cells (nuclei were identified by DAPI-staining). Parental *h+* and *h−* haploids, both of which carried the deletion of interest and only one of which carried the GFP label, were mated and induced to undergo zygotic meiosis. *n=*100; N.D., not determined.

We focused on 18 genes with confirmed phenotypes for further analysis. As a positive control, we used cells lacking Spo11 (also called Rec12 in *S. pombe*), the transesterase responsible for double-strand break formation to initiate meiotic recombination, which show a profound meiotic chromosome segregation defect (Sakuno *et al.* 2011). Five strains failed to generate spores at the normal level and showed phenotypes indicative of defective conjugation (*dbl2Δ*), meiotic entry (*cdt2Δ*, *zds1Δ*), or asci formation (*tpr1Δ*, *dms1Δ*) (representative images are shown in Figure 2A). We selected *SPCC1739.04c* for further study and named this gene *dms1*, for defective meiosis and sporulation (see below). Chromosome segregation was assessed in mutants that performed meiotic divisions by examining the fate of homozygous GFP labels on the arm of chromosome II in mutants that generated spores (Figure 2B). We also examined heterozygous, centromere-linked GFP labels in cells stained with DAPI to visualize the nuclei (Figure 2C). The presence of homozygous GFP labels in one side of the tetrad (two adjacent spores) is suggestive of homolog nondisjunction in meiosis I, while mis-segregation of sister chromatids in meiosis II leads to heterozygous GFP foci within the same spore. All mutants displayed some degree of chromosome mis-segregation, indicative of defective meiosis I and/or meiosis II. Spore viability measurements (Figure S2) largely correlated with the degree of observed chromosome mis-segregation, as expected. As proof of principle, we selected three mutants for further study: *acb1Δ*, *pof3Δ*, and *dms1Δ*, which appear predominantly defective in meiosis I, meiosis II, and sporulation, respectively.

### Acb1 promotes timely sister chromatid separation

**Figure 3:**
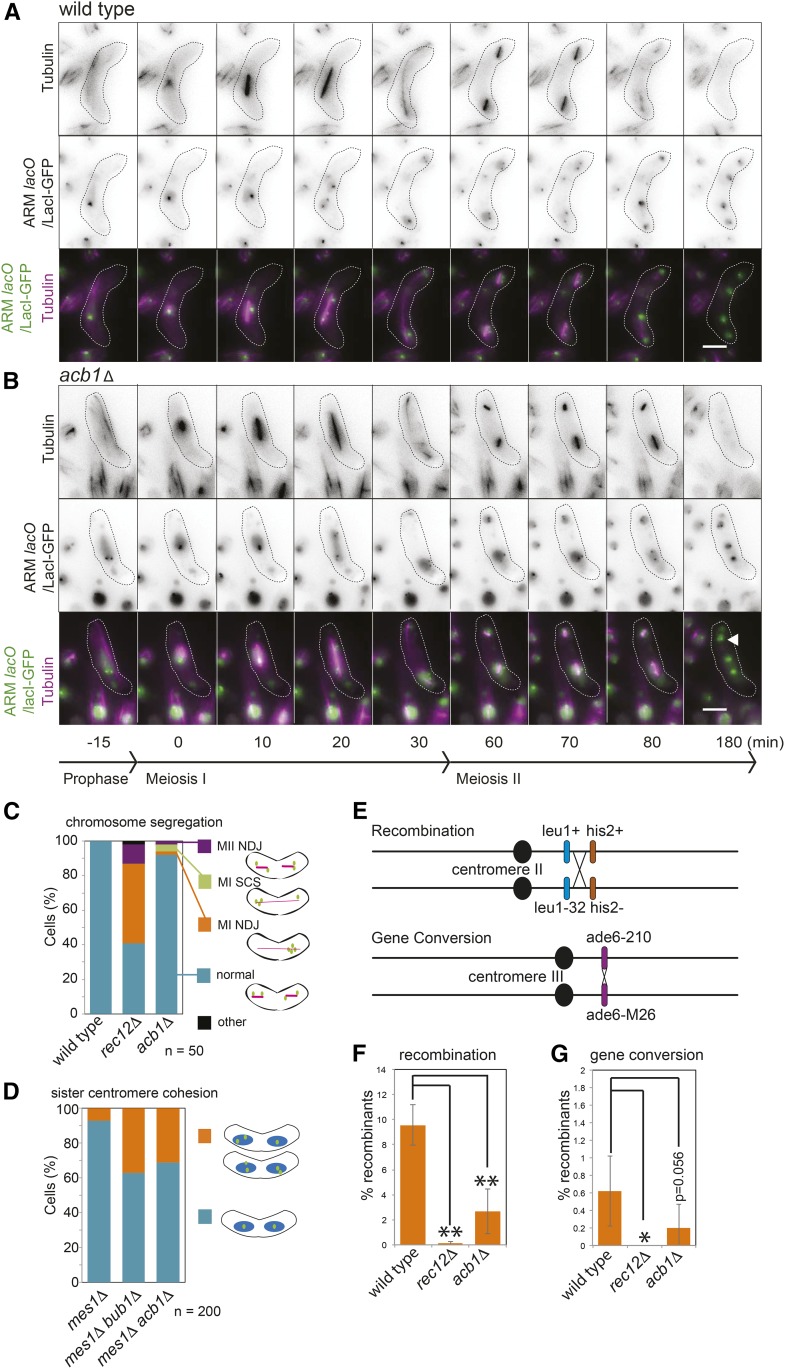
Acb1 is important to prevent premature sister chromatid separation. (A and B) Wild type (AMfy1744) and *acb1Δ* (AMfy1765) cells carrying mCherry-Atb1 *lacO* integrated on the arm of both copies of chromosome II and producing LacI-GFP were induced to undergo zygotic (h^90^) meiosis and imaged at 5-min intervals. Representative still images of wild type (A) and *acb1Δ* (B) cells undergoing meiosis are shown. Arrowhead indicates closely two opposed dots. Bar, 5 μm. (C) Quantification of chromosome segregation errors from movies as in A and B for wild type, *acb1Δ*, and *rec12Δ* (AMfy1703) cells undergoing meiosis. NDJ, nondisjunction; SCS, sister chromatid separation; *n=*50. (D) Analysis of sister chromatid cohesion in *mes1Δ* cells arrested as binucleate cells before meiosis II. Cells were *mes1Δ*, carried *lacO* arrays integrated close to the centromere on both copies of chromosome II, expressed LacI-GFP, and were otherwise wild type (AMfy1786), *bub1Δ* (AMfy1832), or *acb1Δ* (AMfy1797). The indicated patterns of GFP foci were scored in 200 binucleate cells of each strain after inducing zygotic (h^90^) meiosis. (E–G) Meiotic recombination is decreased in *acb1Δ* cells. (E) Schematic of assay showing the positions of markers. (F) Average cross-over recombination frequency during zygotic (h^+^ × h^−^) meiosis for wild type (AMfy1769 × AMfy1778), *rec12Δ* (AMfy1780 × AMfy1794) and *acb1Δ* (AMfy1808 × AMfy1810) is shown. Following meiosis, the percentage of colonies that grew on medium lacking either leucine or histidine (but not both) were scored from a total of 234–1443 colonies for each strain and the average of six biological repeats is shown. Error bars representing SD and *P* values were calculated using *t*-test (** *P* < 0.0001, * *P* < 0.05). (G) Average gene conversion at *ade6* is shown for the experiment described in F.

Acb1/SPBC1539.06 is a predicted fatty-acyl-CoA binding protein, which our preliminary analysis suggested has a modest effect on chromosome segregation (Figure 2, B and C). To examine the chromosome segregation defect caused by the absence of Acb1 more closely, we imaged live wild type, *acb1Δ*, and *rec12Δ* control cells carrying a GFP label on the arm of both copies of chromosome II and mCherry-labeled tubulin undergoing meiosis (Figure 3, A–C). In wild type cells, following the horsetail stage during prophase I, where extensive microtubule-driven movement of the nucleus took place, a short meiosis I spindle formed and extended. Consistent with homologs being segregated at meiosis I, and sister chromatid cosegregation, we observed only one (presumably representing two closely opposed sister chromatids) or two GFP dots moving to opposite poles in wild type cells. Following homolog segregation during meiosis I, two short spindles formed at meiosis II, which elongated to segregate one GFP focus into each of the four resultant nuclei (Figure 3A). In the majority of *acb1Δ* cells, we observed similar behavior to wild type; however, a small fraction of cells exhibited defective segregation. Figure 3B shows an example of meiosis II nondisjunction: two GFP dots segregate to each pole during meiosis I (20 min) but only one pair of these segregate to opposite poles during meiosis II, while the other pair ends up in the same nucleus (Figure 3B, arrowhead). Overall, we observed a low level of mis-segregation in *acb1Δ* cells, including meiosis I sister chromatid separation (MI SCS) where segregation of three GFP labels to a single pole during meiosis I is observed; and nondisjunction of either homologs or sister chromatids at meiosis I (MI NDJ) or II (MII NDJ), respectively (Figure 3C). This modest effect of *acb1Δ* on meiotic chromosome segregation is consistent with our end-point analysis, which also showed a relatively mild phenotype (Figure 2, B and C).

The presence of a small fraction of *acb1Δ* cells where sister chromatids segregated prematurely (during meiosis I) suggested that the underlying defect could affect sister chromatid cohesion. To test this possibility, we arrested cells carrying homozygous centromere-linked LacI-GFP foci in prometaphase II, using the *mes1Δ* mutation (Kitajima *et al.* 2004). At this stage, sister centromere cohesion should be intact so that each nucleus should carry a single GFP dot. However, where sister centromere cohesion is absent or weakened, for example in *bub1Δ* cells (Bernard *et al.* 2001), two GFP dots are more frequently observed within a single nucleus (Figure 3D) (Miyazaki *et al.* 2017). Analysis of the *acb1Δ mes1Δ* mutant revealed ∼30% of cells with separated sister chromatids at prometaphase I, supporting the idea that Acb1 could be important for robust cohesion. The central mediator of sister chromatid cohesion is the cohesin complex. However, we found that the fluorescence intensity of the GFP-tagged cohesin subunit, Rec8, during meiotic prophase, metaphase I, and anaphase I was comparable to wild type (Figure S3, A and B). This suggests that a failure to recruit or maintain sufficient cohesin is not the cause of impaired sister chromatid cohesion in *acb1Δ* cells.

A low level of meiosis I nondisjunction was also observed in *acb1Δ* cells, a potential cause of which is an absence or reduction in chiasmata, as a result of decreased meiotic recombination. We monitored both cross-over recombination and gene conversion in *acb1Δ* cells together with wild type and *rec12Δ* controls using a reporter gene system (Figure 3E). Interestingly, we observed a significant decrease in cross-over recombination in *acb1Δ* cells (Figure 3F). Gene conversion was also decreased in *acb1Δ* cells, though this did not quite reach significance (Figure 3G). These findings suggest that Acb1 may be important for proper levels of meiotic recombination, potentially explaining the low level of meiosis I nondisjunction in *acb1Δ* cells.

To determine the relative levels and localization of Acb1 during meiosis, we tagged the endogenous copy with GFP. Meiotic time course analysis and western blotting indicated that Acb1-GFP was present and localized in the nucleus throughout all stages of meiosis (Figure S3, C–E). We suggest that Acb1 makes a minor contribution to promoting accurate chromosome segregation through meiosis, and may do so by affecting the efficacy of sister chromatid cohesion, a role supported by its localization in the nucleus, though further work is required to test this hypothesis.

### Pof3 is critical for meiosis I and meiosis II chromosome segregation

**Figure 4:**
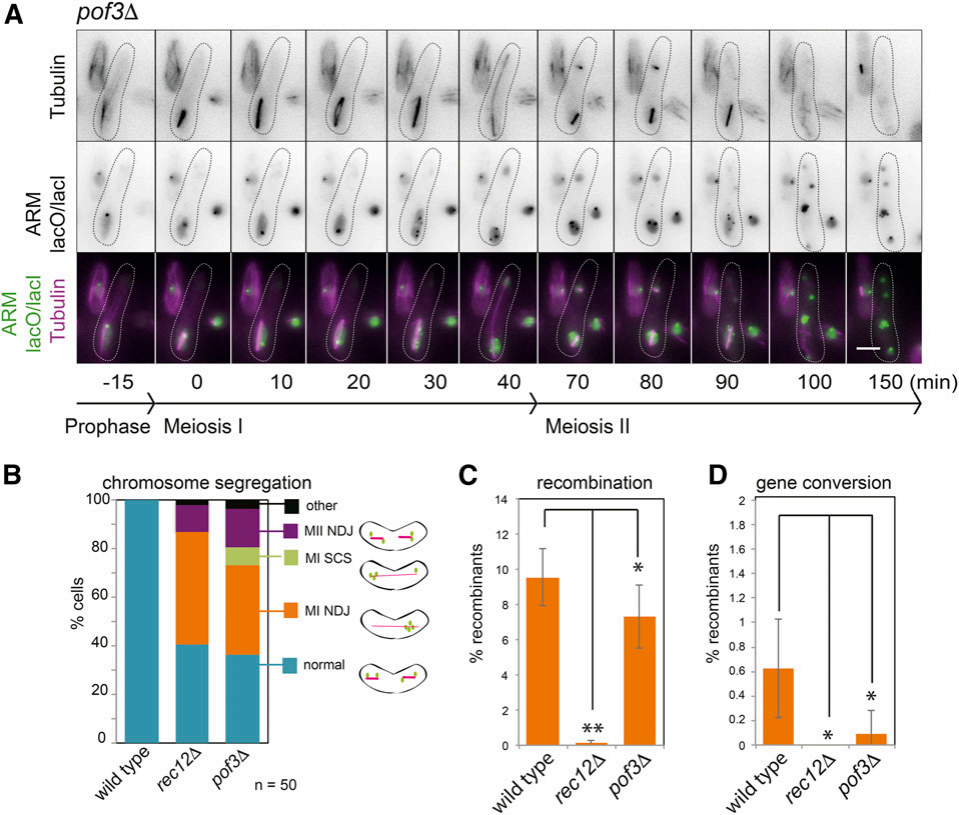
Pof3 is critical for accurate chromosome segregation during meiosis I and meiosis II. (A) Representative images from a movie of *pof3Δ* zygotic (h^90^) meiosis carrying mCherry-Atb1, *lacO* integrated on the arm of both copies of chromosome II, and producing LacI-GFP and imaged as described in Figure 3 (strain AMfy1827). (B) Quantification of chromosome segregation errors from movies. Data for wild type and *rec12Δ* is reproduced from Figure 3C. Cross-over recombination (C) and gene conversion (D) were assayed as described in Figure 3, E–G. The data for wild type and *rec12Δ* is reproduced from Figure 3, F and G. The data for *pof3Δ* was generated via a cross between strains AMfy1865 and AMfy1866. Error bars representing SD and *P* values were calculated using *t*-test (** *P* < 0.0001, * *P* < 0.05).

Pof3 is a so-called F box protein, a family of proteins that confer substrate specificity to the Skp1-Cdc53/Cullin-1-F box (SCF) ubiquitin ligase, which marks targets for degradation (Katayama *et al.* 2002; Petroski and Deshaies 2005). Though viable, *pof3Δ* cells show defective centromere function in mitosis (Takayama *et al.* 2010) and exhibited a profound chromosome segregation defect in our screen and preliminary characterization (Figure 2, B and C). Live cell imaging of *pof3Δ* cells carrying a GFP label on chromosome arms and mCherry-tubulin revealed meiosis I nondisjunction, that is a failure of homologs to segregate to opposite nuclei, to be the predominant error, approaching the expectation for completely random homolog segregation (50%) (Figure 4, A and B: MI NDJ). In addition, a substantial fraction of cells exhibited erroneous sister chromatid segregation (Figure 4B): either premature sister chromatid separation and segregation during meiosis I (MI SCS), or sister chromatid nondisjunction during meiosis II (MII NDJ). In the example shown (Figure 4A; scored as MI NDJ in Figure 4B), both meiosis I and meiosis II nondisjunction occurs: all four GFP foci initially segregate to the same pole during meiosis I (40 min) and subsequently, during meiosis II, one of these foci is segregated away from the other three (arrowhead). Therefore, Pof3 may be important for multiple processes that impinge on chromosome segregation. The nondisjunction of sister chromatids raised the possibility that centromeric cohesion was defective in *pof3Δ* mutants. Indeed, sister centromeres were moderately more separated in *pof3Δ* prometaphase II-arrested cells (*mes1Δ* background; Figure S4A). However, this does not appear to be due to premature loss of Rec8 cohesin from centromeres; rather fluorescence intensity measurements suggested elevated Rec8 levels on chromosomes in *pof3Δ* mutants (Figure S4B). We also observed a modest, though significant, reduction in both meiotic cross-overs and gene conversion in *pof3Δ* cells (Figure 4, C and D). These findings suggest a role for Pof3 in both cohesion and recombination, raising the possibility that cohesin, which contributes to both of these processes, may be a key target of Pof3-SCF; however, we cannot exclude other possibilities (see *Discussion*).

### Dms1/SPCC1739.04c: a new spindle pole body regulator

**Figure 5:**
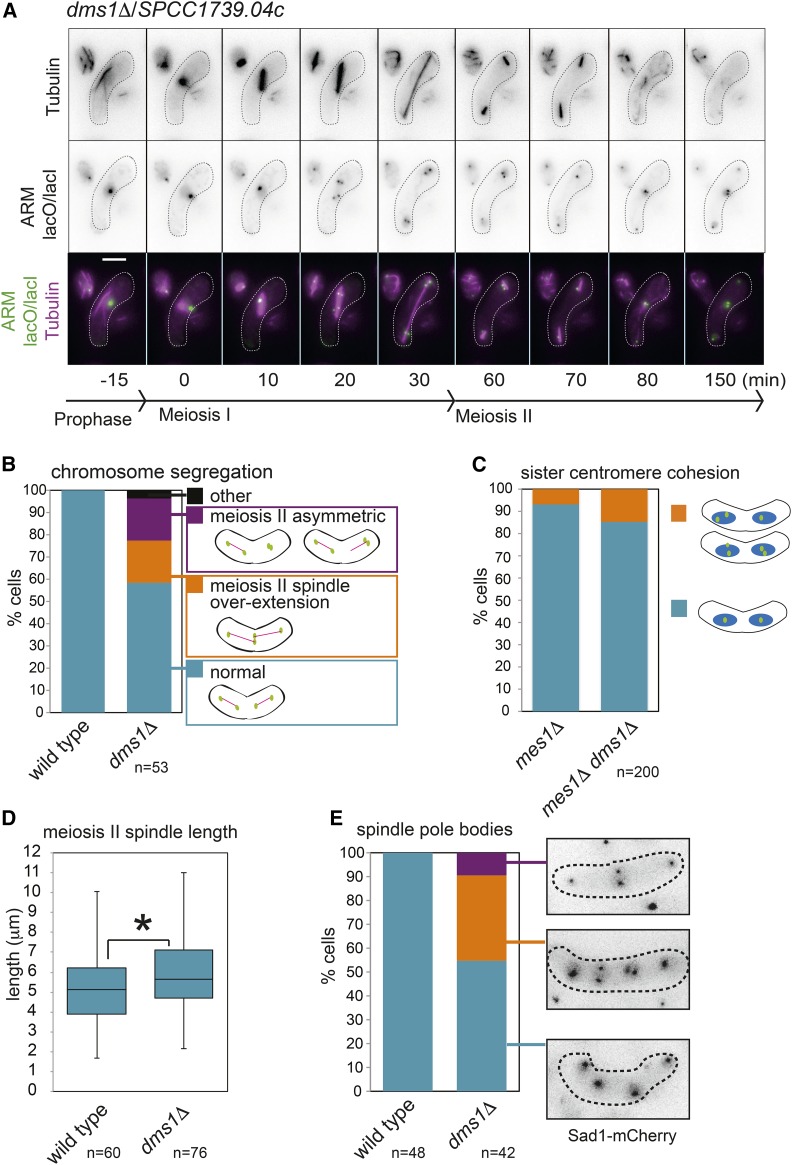
Dms1 is important for SPB function in *S. pombe* meiosis. (A) Representative images from a movie of *dms1Δ* cells carrying mCherry-Atb1, *lacO* integrated on the arm of both copies of chromosome II and producing LacI-GFP imaged as described in Figure 3 (strain AMfy1553). (B) Quantification of phenotypes observed in *dms1Δ* cells in movies of meiosis as in A. (C) Analysis of sister chromatid cohesion in *mes1Δ dms1Δ* cells (AMfy1867) arrested as binucleate cells before meiosis II as described in Figure 3D. Data for *mes1Δ* are reproduced from Figure 3D. (D) The longest meiosis II spindle length observed was measured in at least 60 cells for wild type and *dms1Δ* cells from movies as in A. * *P* = 0.0177. (E) The number and position of SPBs was scored from movies of wild type (AMfy1098) and *dms1Δ* (AMfy1112) cells carrying Sad1-mCherry and undergoing meiosis II.

SPCC1739.04c is a *Schizosaccharomyces*-specific protein that was also found to have ascospore formation defects in two previous screens (Gregan *et al.* 2005; Ucisik-Akkaya *et al.* 2014). We named this gene *dms1* for *defective meiosis and sporulation* as we discovered that it is also important earlier in meiosis (see below). Live cell imaging of *dms1Δ* cells carrying LacI-GFP labels on the arm of both copies of chromosome II and mCherry-tagged tubulin revealed an unprecedented phenotype (Figure 5A). Meiosis I chromosome segregation proceeded normally, with one (or two closely opposed) GFP foci segregating to each pole; subsequently, and also as expected, single GFP foci segregated to opposite poles on meiosis II spindles. In late anaphase II, however, the two spindles elongated and met in the center of the cell with the result that two of the chromatids (GFP foci) come into very close proximity, rather than being spaced evenly throughout the cell. This unusual phenotype was observed in ∼20% of cells. In a further ∼20% of cells, meiosis II occurred in an asymmetric manner: that is one of the nuclei built a spindle and segregated sister chromatids, while the other one failed to do so (Figure 5B). Consistent with the idea that chromosome segregation proceeds normally until anaphase II, sister chromatids were essentially normally cohered during prometaphase II (Figure 5C). Instead, spindle function appeared defective in these cells. We found that meiosis II spindle length was significantly increased in *dms1Δ* cells (Figure 5D) though the time spent in meiosis, or meiosis II specifically, was unchanged compared to wild type (Figure S5A). Increased meiosis II spindle length was particularly evident in the fraction of cells where spindles were seen to overlap (Figure S5B), providing an explanation for this phenomenon. Consistent with increased meiosis I spindle length, visualization of spindle pole bodies (SPBs) in *dms1Δ* cells carrying Sad1-mCherry identified a fraction of cells in which two SPBs were closely juxtaposed at the cell center. Moreover, we observed in excess of four SPBs in more than a third of *dms1Δ* meiosis II cells (Figure 5E). These findings indicate that Dms1 plays an important role in regulating spindle length, potentially through influencing SPB number and position.

**Figure 6:**
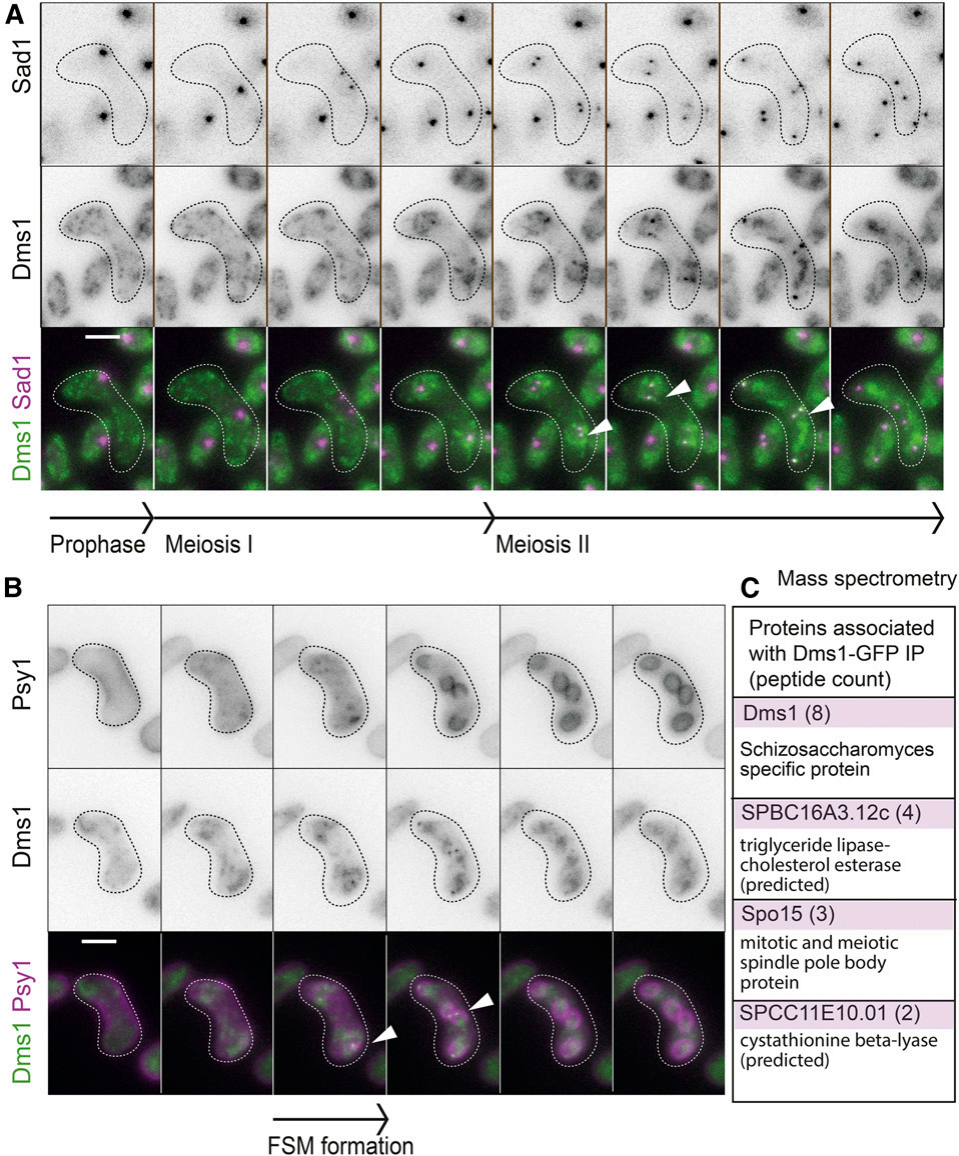
Dms1 associates with SPBs during meiosis II. (A) Live cell imaging of Dms1 and SPBs during meiosis. Strain AMfy1845 carrying Dms1-GFP and Sad1-mCherry was induced to undergo zygotic (h^90^) meiosis and images captured at 5-min intervals. Representative images from a movie are shown. Bar, 5 μm. (B) Representative images from live cell imaging showing forespore membrane (FSM; mCherry-Psy1) and Dms1 (Dms1-GFP) localization (AMfy1807). (C) Proteins interacting with Dms1 in meiosis. Diploid strains AMfy1796 (Dms1-GFP) and AMfy781 (no tag control) were harvested 7 hr after induction of azygotic synchronous meiosis and anti-GFP immunoprecipitates were analyzed by mass spectrometry. Proteins found to be specific to the Dms1-GFP sample and where two or more peptides were identified are shown.

Examination of GFP-tagged endogenous Dms1 during synchronous meiosis by western blotting revealed that it is present at all stages of meiosis (Figure S6, A and B). Interestingly, a high molecular weight band appeared coincident with the onset of nuclear divisions and reached maximal intensity at 24 hr, at which time ascospores are produced. This suggests that Dms1 may be modified late in meiosis and that this could play a role in ascospore formation though the identity of this modification remains unknown. Imaging of Dms1-GFP cells revealed punctate foci throughout the cell at all stages of meiosis. However, we noticed that, particularly in late meiosis, some of the puncta tended to associate in a pattern that was reminiscent of the forespore membrane (Figure S6C). Given this localization pattern, and the effect of *dms1Δ* on SPB behavior, we tested for Dms1-GFP colocalization with SPBs and forespore membranes. Diploid strains carrying Dms1-GFP and Sad1-mCherry (to mark SPBs) were imaged as cells underwent meiosis. Interestingly, we observed Dms1-GFP on SPBs specifically during meiosis II (Figure 6A; arrowheads). Labeling of the prospore membrane (Psy1-mCherry) together with Dms1-GFP did not reveal a strict colocalization; however, the Psy1-mCherry signal tended to partially overlap meiosis II-specific Dms1-GFP foci, likely corresponding to the SPBs. To identify potential interactors of Dms1, we purified Dms1-GFP from cells harvested 7 hr after induction of synchronous meiosis and analyzed immunoprecipitates by mass spectrometry. Interestingly, Spo15, which localizes to the SPB and is required for spore membrane formation (Ikemoto *et al.* 2000) copurified with Dms1-GFP (Figure 6C and Table S7 in File S1).

**Figure 7:**
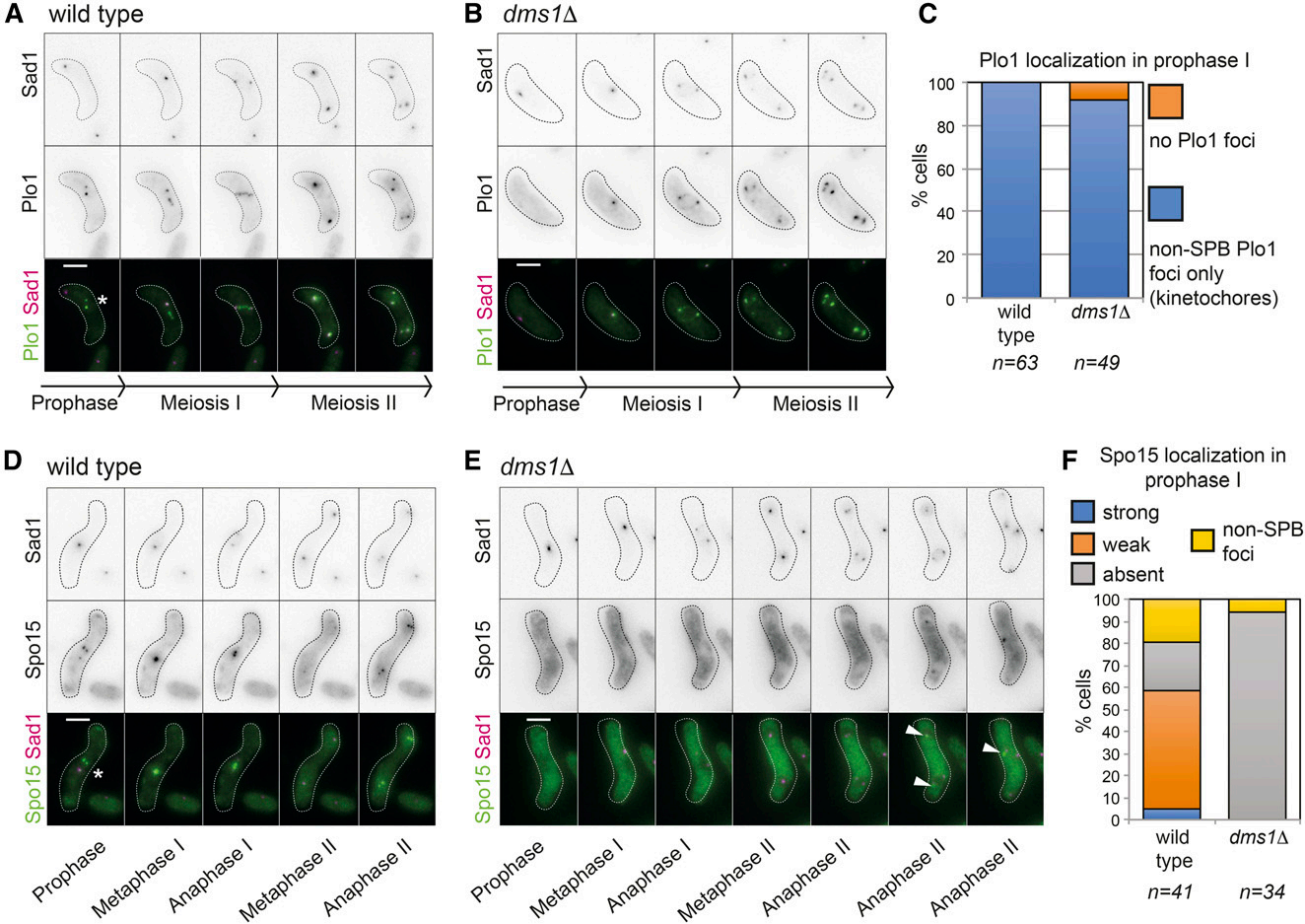
Dms1 regulates Spo15 and Plo1 localization during meiosis I. (A–C) Plo1 dissociates from the SPB during prophase I in *dms1Δ* cells and forms foci consistent with localization to kinetochores in the majority of cells. Representative images from movies of zygotic (h^90^) meiosis are shown for wild-type (A; AMfy1847) and a *dms1Δ* (B, AMfy1859) cell where presumptive kinetochore localization was not detected. Both strains carry Plo1-GFP and Sad1-mCherry. Bar, 5 μm. Asterisk in A indicates presumed kinetochore foci. (C) Quantification of Plo1 localization during prophase is shown. (D–F) Dms1 is required for Spo15 localization at SPBs during meiosis I. Live cell imaging of Spo15-GFP in wild type (D; AMfy1854) and *dms1Δ* (E; AMfy1860) cells undergoing zygotic (h^90^) meiosis and also carrying Sad1-mCherry. Asterisk in D indicates presumed kinetochore foci. Arrowheads in E indicate Spo15-GFP at SPBs in meiosis II. (F) Scoring of Spo15-GFP foci in prophase I is shown.

SPBs undergo remodeling during meiosis dependent on *S. pombe* Polo kinase, Plo1, which is excluded from the SPB during meiotic prophase I, and localizes to kinetochores instead (Krapp *et al.* 2010; Funaya *et al.* 2012; Ohta *et al.* 2012). Forced Plo1 maintenance on the SPB during prophase I leads to recruitment of factors that are normally delocalized, and SPB overduplication (reminiscent of the *dms1Δ* phenotype; Figure 5E). Therefore, exclusion of Plo1 and associated factors, among them Spo15, is important for SPB remodeling. To test whether Dms1 contributes to SPB remodeling, we examined Plo1 localization in *dms1Δ* cells and found that, as in wild type cells, Plo1-GFP was excluded from the SPB (no colocalization with Sad1-mCherry) during prophase I, but was recruited to the SPB from meiosis I until meiotic exit (Figure 7, A and B). Two non-SPB foci of Plo1-GFP, presumed to be the kinetochores (Krapp *et al.* 2010; Ohta *et al.* 2012), were also observed in wild type (Figure 7A, asterisk) and the majority, though not all (*e.g.*, Figure 7B), *dms1Δ* prophase I cells (Figure 7C). Therefore, we find no strong evidence that Dms1 influences SPB remodeling or chromosome segregation through affecting Plo1 localization. To examine the relationship between Dms1 and its interacting partner Spo15 (Figure 6C), we imaged wild type and *dms1Δ* cells carrying Spo15-GFP. A previous live cell imaging analysis failed to detect Spo15-GFP at the SPB during prophase I (Ohta *et al.* 2012), though a prior immunofluorescence study did find Spo15 associated with the SPB (Ikemoto *et al.* 2000). This is likely due to the sensitivity of the imaging conditions as in our hands, during prophase I, Spo15-GFP was diminished at, but not absent from, the SPB in wild type cells (Figure 7D). Interestingly, two further non-SPB Spo15 foci were detected in prophase I wild type cells, raising the interesting possibility that Spo15 associates with kinetochores, in addition to SPBs (Figure 7D, asterisk). In *dms1Δ* cells, Spo15 was largely delocalized, although residual signal was detected at SPBs in meiosis II (Figure 7E, arrowheads and Figure 7F). Therefore, it is likely that Dms1 regulates the localization of Spo15, thereby remodeling the SPB to ensure proper meiotic spindle behavior and spore formation.

## Discussion

### Novel meiosis and sporulation genes

We present a comprehensive analysis of virtually all nonessential *S. pombe* genes in meiosis and sporulation. Overall, we found that, though not essential for vegetative growth, >10% of nonessential *S. pombe* genes are important for efficient conjugation or sporulation and a further ∼8% are important for proper meiotic chromosome segregation. Most intriguingly, ∼30 genes in each category are uncharacterized and could take on novel functions during meiosis. While some of these genes appear widely conserved, recognizable orthologs are apparent only in *Schizosaccharomyces* species for a large fraction of the genes. We note that the cohesin protector Mei-S332 was long-known in *Drosophila* (Kerrebrock *et al.* 1995), and was not thought to be conserved, until genetic screens in yeast identified shugoshins as distant homologs (Kitajima *et al.* 2004). Similarly, the recently identified meiosis I-specific Meikin protein is thought to be a distant homolog of *S. pombe* Moa1 and the long-known *S. cerevisiae* meiosis I-regulator Spo13, based on similar functions and binding interactions, though no homology can be detected between these proteins based on sequence alone (Klapholz and Esposito 1980; Kim *et al.* 2015). We anticipate that, similarly, functional studies will reveal conserved roles for the newly identified regulators.

Our study implicated a greater percentage of the genome in sporulation compared to a previous screen, which found a requirement for only ∼1% of the genome in sporulation (Ucisik-Akkaya *et al.* 2014). This difference is presumably due to different screening methods. The previous screen relied on the absence of cell walls in sporulation-defective mutants, which results in reduced staining by iodine (Ucisik-Akkaya *et al.* 2014), while our screen individually analyzed each mutant microscopically. This allowed us to be less stringent in our criteria (<20% spore formation) and include genes important for efficient sporulation, in addition to genes essential for sporulation.

### Genes with central roles in metabolism, protein homeostasis, and spindle pole body function are important for meiosis

We found that Acb1 is important for accurate chromosome segregation and potentially ensures robust sister chromatid cohesion. Acb1 is a member of a broadly conserved large family of acyl-coA-binding proteins and may thus play a role in lipid homeostasis (Neess *et al.* 2015). We note that although lipid metabolism normally occurs in the cytoplasm, we found that Acb1 is localized in the nucleus during meiosis, suggesting additional roles (Figure S3C). Although it is possible that Acb1 plays a “moonlighting” role in chromosome segregation, we favor the idea that *acb1Δ* has an indirect effect, arising from changes in acyl-CoA metabolism. We speculate that, through sequestration of acyl-CoA esters and supporting their associated onward metabolism, Acb1 affects the intracellular concentrations of coenzyme A. This is a central metabolite in diverse cellular biochemistry including protein acetylation, which is of key importance in chromosome function (Galdieri *et al.* 2014; Pietrocola *et al.* 2015). It is conceivable that alterations in histone acetylation account for the observed phenotypes in *acb1Δ* cells, that is, defective centromeric cohesion and reduced meiotic recombination. Another substrate of interest is cohesin itself, since acetylation of its Smc3 subunit (Psm3 in *S. pombe*) is known to be important for cohesion establishment (Feytout *et al.* 2011).

Pof3 is an F box protein that confers substrate specificity to the SCF ubiquitin ligase, the role of which is to target proteins for degradation (Deshaies 1999). Previous work showed that the Ams2 transcription factor, which is important for histone expression, is a key SCF^Pof3^ target (Chen *et al.* 2003; Takayama *et al.* 2010). Excess Ams2 has been found to increase histone levels and thereby interfere with assembly of the centromere-specific histone variant CENP-A, negatively affecting centromere function. Therefore, defective centromere function could explain why loss of Pof3 drastically affected chromosome segregation during both meiosis I and II.

Cells lacking *dms1* presented with an unusual phenotype in which the innermost two nuclei in fraction of tetrads met during meiosis II. We traced this phenotype to overelongation of the meiosis II spindle, likely mediated by a defect in SPBs regulation. How SPBs, and thereby nuclei, are evenly spaced within the differentiating cell is not known. Identification of Dms1 as a mediator of this spacing will inform future studies into this question. Given that we found Dms1 to be associated with the SPB during meiosis II, it is tempting to speculate that a Dms1-dependent signal from the SPB sets up a zone of mutual inhibition for spindle elongation. Dms1 is required for proper localization of its interacting partner, Spo15, at SPBs. Spo15 is required for prospore membrane formation (Ikemoto *et al.* 2000; Nakase *et al.* 2008; Ohta *et al.* 2012) and, although not tested directly, our data are consistent with Dms1 sharing this function. Therefore, we propose that Dms1 works together with Spo15 to modify SPBs for spore formation during meiosis II, akin to meiosis II-specific modification of the outer SPB plaque in budding yeast (Neiman 2011).

### A comprehensive data set to inform our understanding of meiosis

Our genome-wide screen can be exploited to shed light on the molecular mechanisms underlying meiosis and gamete formation. Through analysis of specific genes, we have demonstrated the potential of this approach to understand how diverse cellular processes affect meiosis. Importantly, our data set includes many as-yet-uncharacterized genes to which functions have not been assigned, potentially reflecting the paucity of studies that assess the requirement for gene function during meiosis and gamete differentiation. Analysis of these genes is a priority for future study.

## Supplementary Material

Supplemental material is available online at www.genetics.org/lookup/suppl/doi:10.1534/genetics.117.300527/-/DC1.
