# Supplementary figures and images for: Genes Important for Schizosaccharomyces pombe Meiosis Identified Through a Functional Genomics Screen
Source: Genetics. 2017 Dec 19;208(2):589–603. doi: 10.1534/genetics.117.300527 (PMC5788524; doi:10.1534/genetics.117.300527)

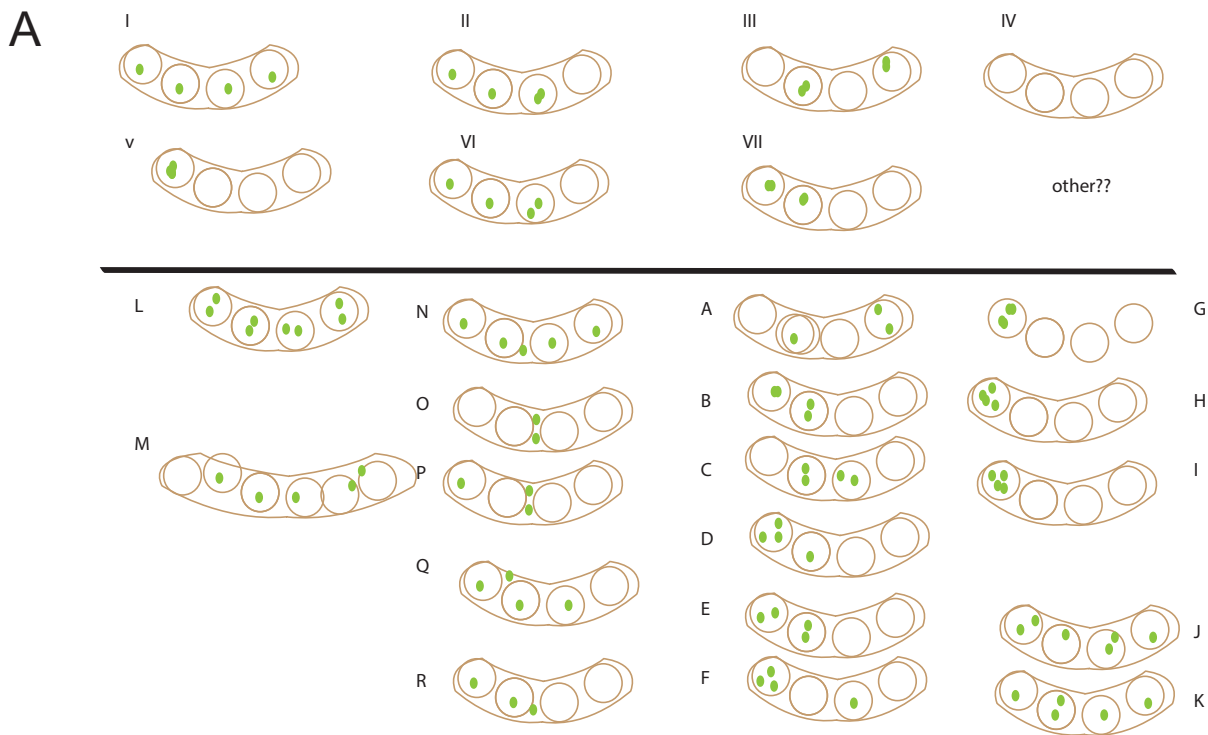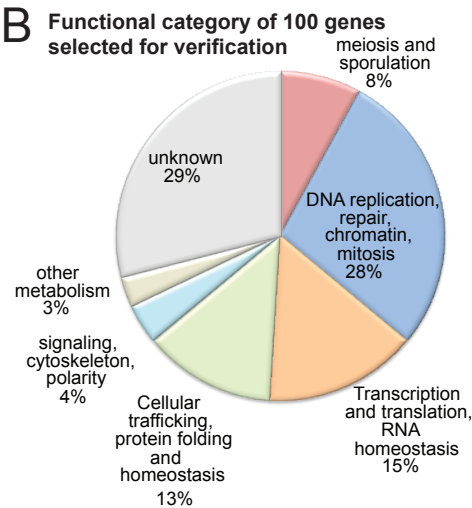

**C Phenotype of 100 mutants in original screen**

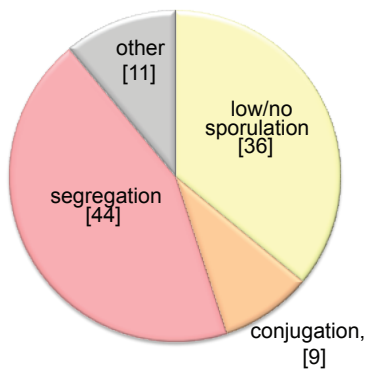

**D Phenotype of 100 mutants after verification**

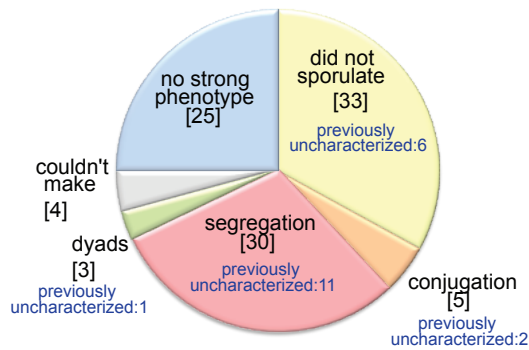

Supplement: Supplementary file 1 [file 589FigureS1.pdf]

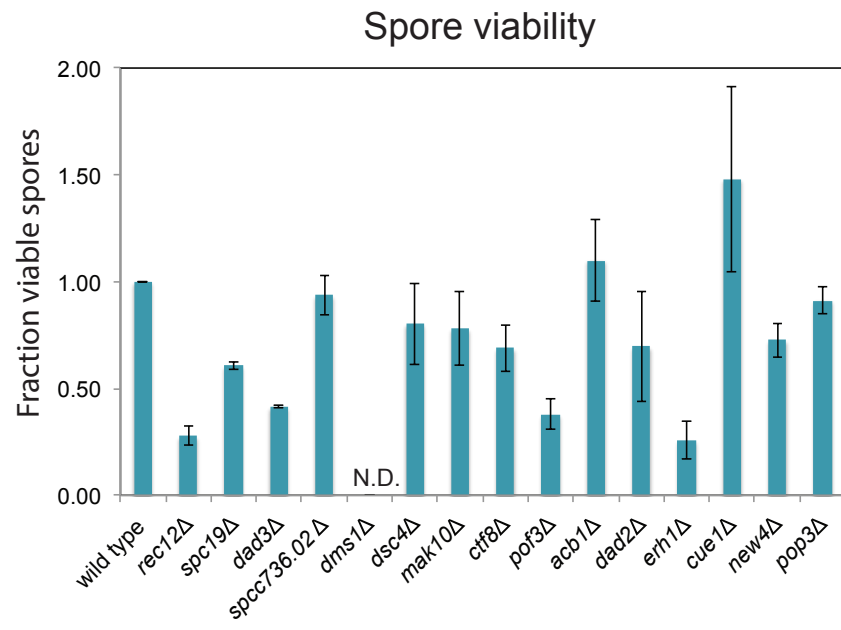

Supplement: Supplementary file 2 [file 589FigureS2.pdf]

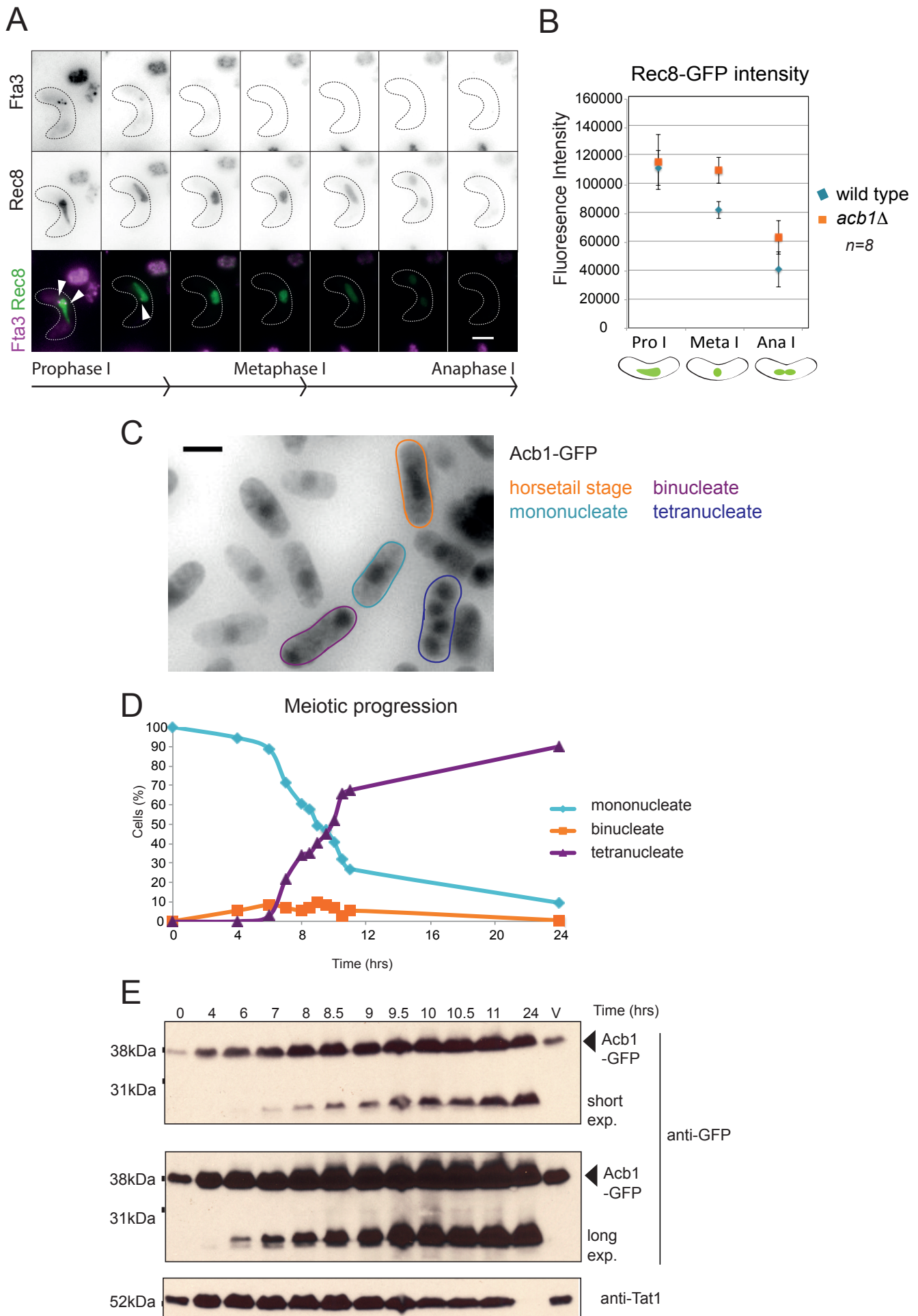

Supplement: Supplementary file 3 [file 589FigureS3.pdf]

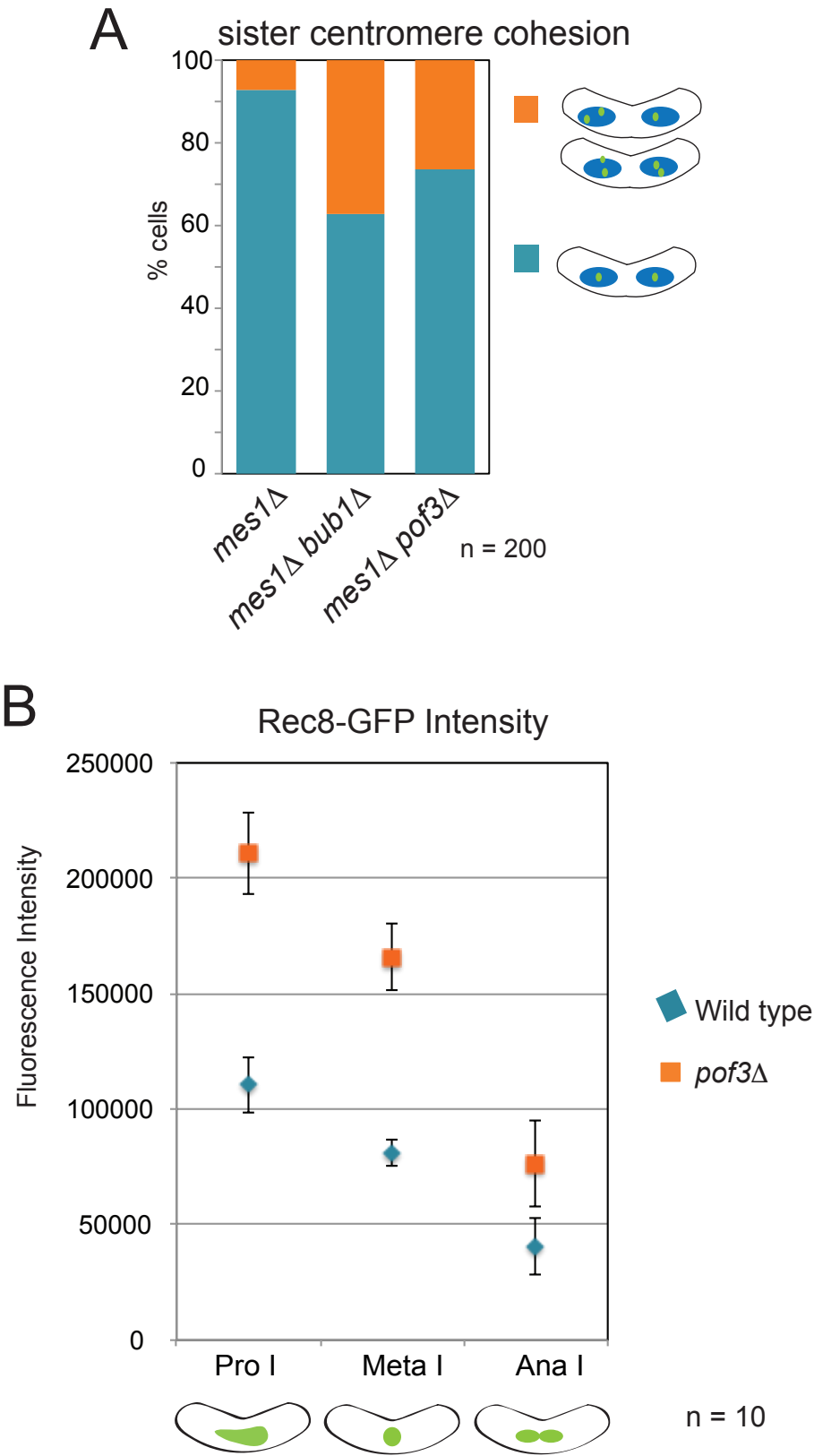

Supplement: Supplementary file 4 [file 589FigureS4.pdf]

A

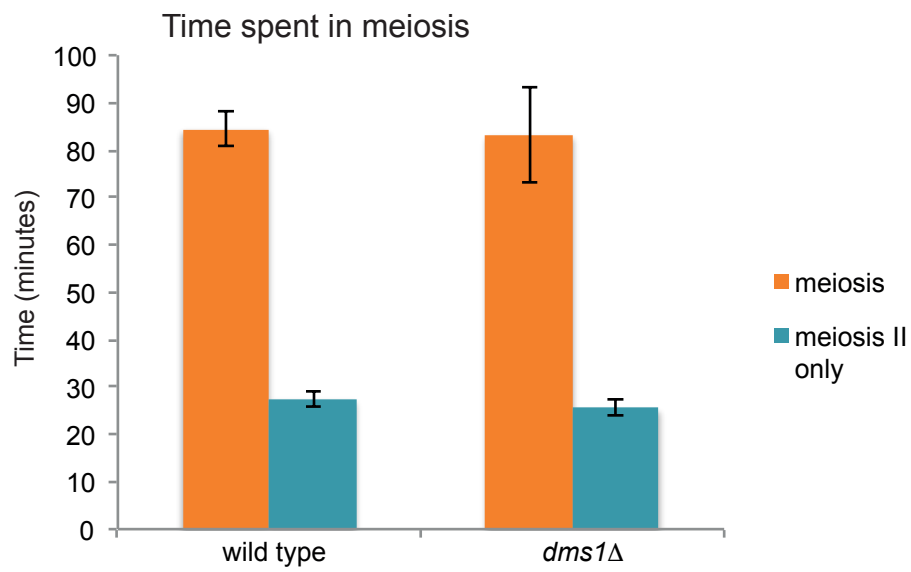

B

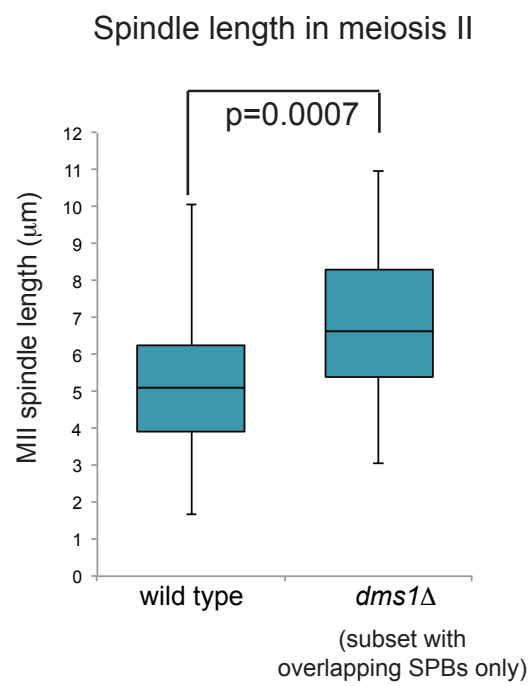

Supplement: Supplementary file 5 [file 589FigureS5.pdf]

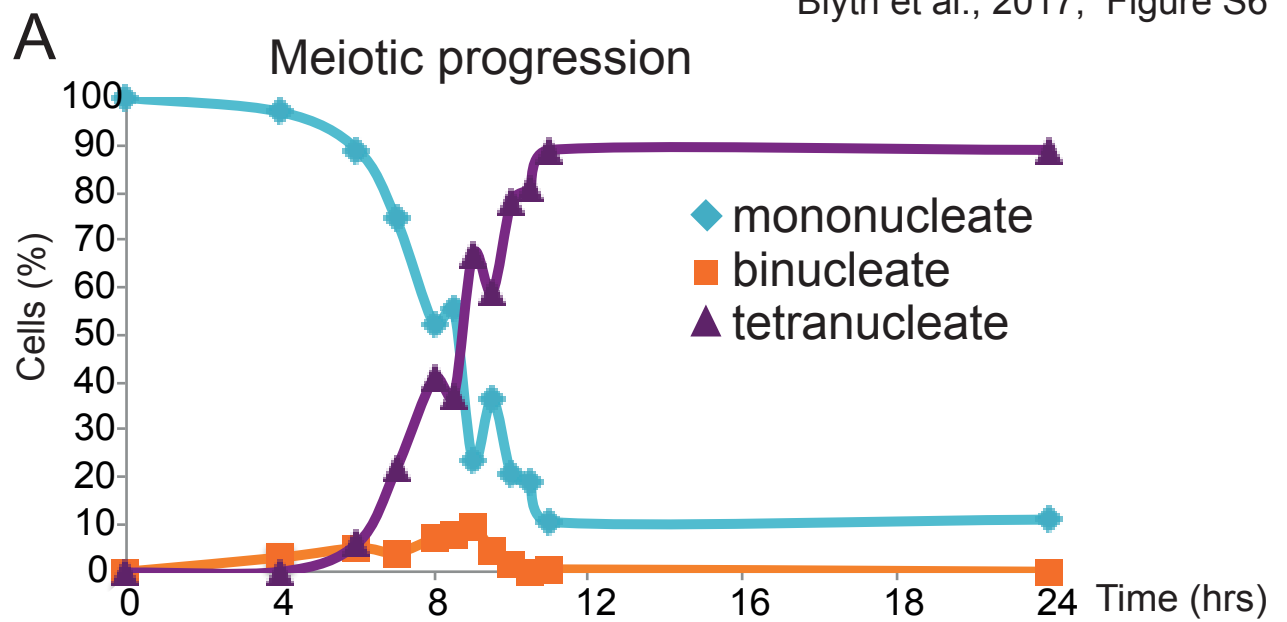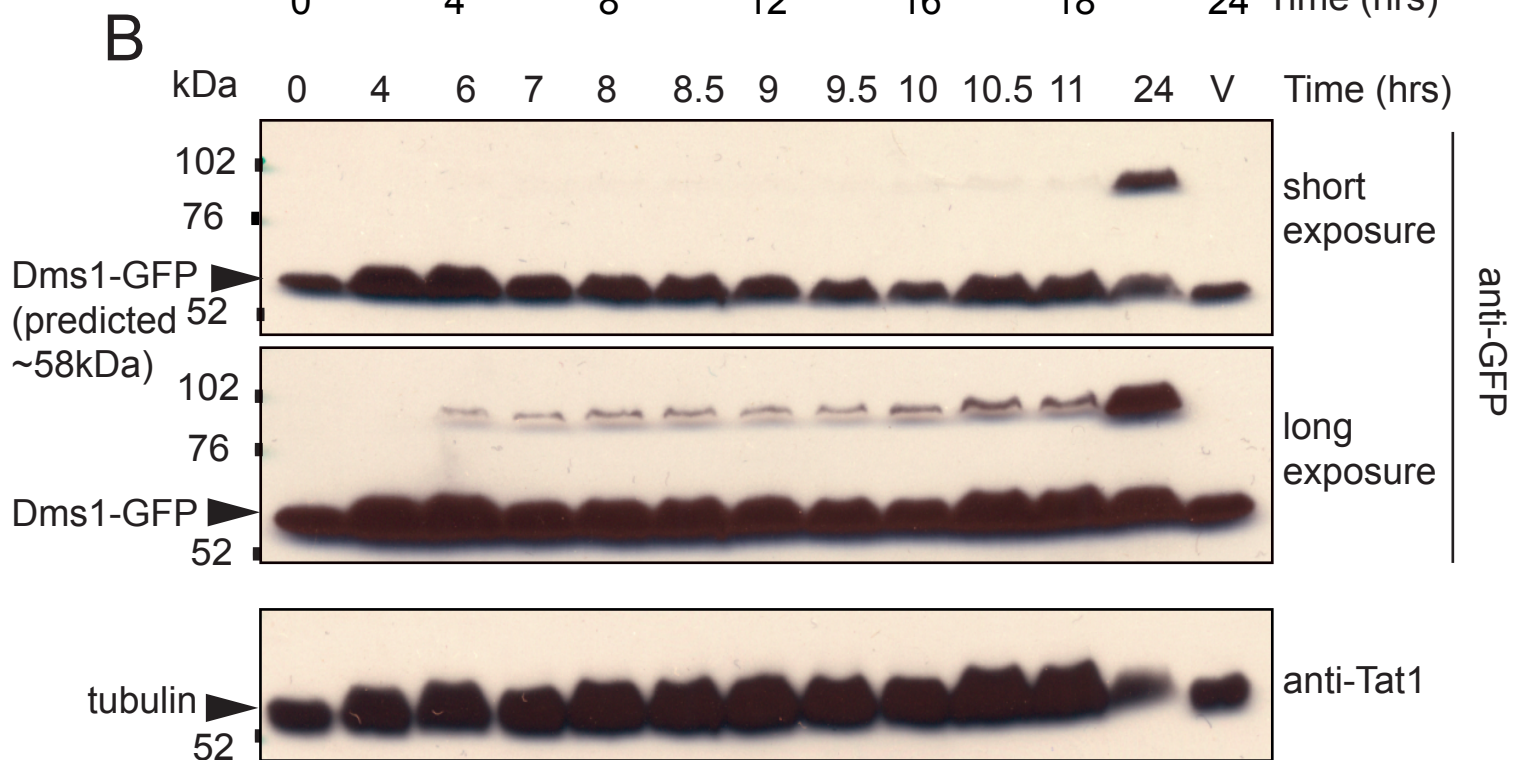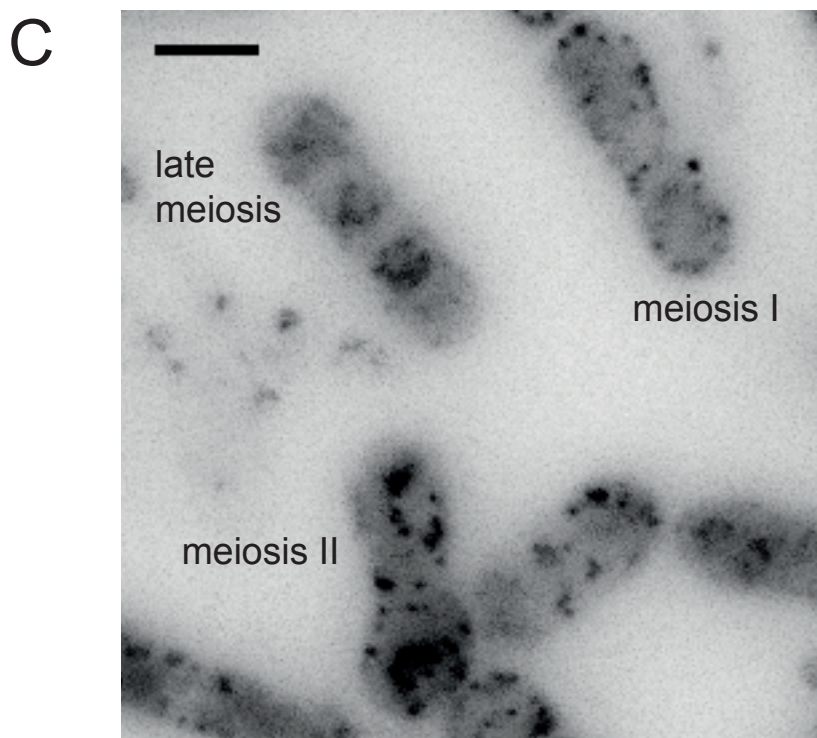

Supplement: Supplementary file 6 [file 589FigureS6.pdf]
